# Virtual reality exposure therapy as treatment for pain catastrophizing in fibromyalgia patients: proof-of-concept study (Study Protocol)

**DOI:** 10.1186/1471-2474-12-85

**Published:** 2011-04-30

**Authors:** Linzette D Morris, Karen A Grimmer-Somers, Bruce Spottiswoode, Quinette A Louw

**Affiliations:** 1 Division of Physiotherapy, Faculty of Health Sciences, Stellenbosch University, Tygerberg, 7505, South Africa; 2 Centre for Allied Health Evidence, University of South Australia, Adelaide, Australia; 3 MRC/UCT Medical Imaging Research Unit, Department of Human Biology, University of Cape Town and Department of Radiology, Stellenbosch University, Tygerberg, South Africa

**Keywords:** *fibromyalgia syndrome*, *pain catastrophizing*, *virtual reality exposure therapy*, *exercise and compliance*

## Abstract

**Background:**

Albeit exercise is currently advocated as one of the most effective management strategies for fibromyalgia syndrome (FMS); the implementation of exercise as a FMS treatment in reality is significantly hampered by patients' poor compliance. The inference that pain catastrophizing is a key predictor of poor compliance in FMS patients, justifies considering the alteration of pain catastrophizing in improving compliance towards exercises in FMS patients. The aim of this study is to provide proof-of-concept for the development and testing of a novel virtual reality exposure therapy (VRET) program as treatment for exercise-related pain catastrophizing in FMS patients.

**Methods:**

Two interlinked experimental studies will be conducted. Study 1 aims to objectively ascertain if neurophysiological changes occur in the functional brain areas associated with pain catastrophizing, when catastrophizing FMS subjects are exposed to visuals of exercise activities. Study 2 aims to ascertain the preliminary efficacy and feasibility of exposure to visuals of exercise activities as a treatment for exercise-related pain catastrophizing in FMS subjects. Twenty subjects will be selected from a group of FMS patients attending the Tygerberg Hospital in Cape Town, South Africa and randomly allocated to either the **VRET **(intervention) group or **waiting list **(control) group. Baseline neurophysiological activity for subjects will be collected in study 1 using functional magnetic resonance imaging (fMRI). In study 2, clinical improvement in pain catastrophizing will be measured using fMRI (objective) and the pain catastrophizing scale (subjective).

**Discussion:**

The premise is if exposing FMS patients to visuals of various exercise activities trigger the functional brain areas associated with pain catastrophizing; then as a treatment, repeated exposure to visuals of the exercise activities using a VRET program could possibly decrease exercise-related pain catastrophizing in FMS patients. Proof-of-concept will either be established or negated. The results of this project are envisaged to revolutionize FMS and pain catastrophizing research and in the future, assist health professionals and FMS patients in reducing despondency regarding FMS management.

**Trial registration:**

PACTR201011000264179

## Background

Fibromyalgia syndrome (FMS) is arguably one of the most complex chronic pain conditions to manage [1]. Characterized by widespread musculoskeletal symptoms and functional disability; FMS is a debilitating condition which affects millions of people around the world [1]. The significant impact FMS has on quality of life, society, industry, healthcare systems and state budgets has rendered FMS a global public health concern [2,3].

Albeit the recent recognition of FMS as a legitimate condition, the exact aetiology of FMS remains unknown [1,4]. Finding effective management strategies for FMS thus continues to frustrate patients and health professionals [4,5]. Largely based on symptomatic relief, the management of FMS usually consists of a combination of pharmacological and non-pharmacological therapies [4,5]. With no one intervention found to be superiorly more effective than the next, FMS management programs are typically individualized to the patient's needs and progressed accordingly [4,5]. Nonetheless, since most FMS patients display some degree of functional disability and muscle deconditioning, the current international consensus is that exercise therapy should at least be a key component of most FMS management programs [6,7]. Evidence from clinical trials has reported that exercise therapy can be beneficial to most FMS patients and is effective in reducing functional disability and FMS symptoms [8,9]. For this reason, exercise therapy is currently one of the most advocated management strategies for FMS [9,10].

The implementation of exercise therapy as a management strategy for FMS in reality is however significantly hampered by poor compliance to exercise programs among FMS patients [11]. Poor compliance towards exercise and other treatments is a common trait among FMS patients and is the primary factor contributing to the chronicity and accelerated deterioration of the condition [11]. A key predictor of poor compliance towards exercise or activity has recently been identified as pain catastrophizing; a cognitive strategy broadly defined as "an exaggerated negative orientation towards actual or anticipated pain experiences" which significantly contributes to the maintenance of chronic pain [12,13].

Currently, the role of pain catastrophizing is believed to be more pronounced in FMS than in other rheumatologic conditions and is recognized as a barrier to the healthy development of psychological and physical functioning among FMS patients [13,14]. Of concern is that in FMS patients, the presence of pain catastrophizing leads to fear-avoidance behaviours which often results in attrition of physical activity [14]. Inactivity is particularly detrimental in FMS and typically leads to further complications such as weakening of the musculoskeletal system, increased pain, increased fatigue and functional disability [8-10]. To date, research has focused on identifying the predictors of poor treatment compliance in FMS, but to our knowledge there is no research into management approaches aimed at addressing these predictors and poor compliance to exercise programs in FMS. The inference that pain catastrophizing and subsequent fear-avoidance behaviours may influence the compliance of FMS patients towards exercise programs, justifies considering the alteration of pain catastrophizing in the management of FMS [14,15].

Evidence suggests that cognitive-behavioural therapy, specifically exposure therapy, may be useful in the alteration of pain catastrophizing [15-18]. Exposure therapy involves exposing an individual to a task or movement that he/she fears, until the emotion of fear is alleviated and disassociated from that particular task or movement [19]. In improving compliance towards exercise programs in FMS, the use of exposure therapy is based on the hypothesis that if patients are repeatedly exposed to visuals of exercise activities or situations that they report to catastrophize, pain catastrophizing levels should decrease over time. The decrease in pain catastrophizing should ideally lead to a decrease in fear-avoidance behaviours, which could fundamentally increase compliance to the recommended exercise programs. With improved compliance to exercise programs, FMS patients may be able to realize the true benefits of exercise and enjoy an improved quality of life.

Although exposure therapy is traditionally conducted during real situations (in vivo exposure therapy) or during an imagined situation (imagined exposure therapy), more recent innovations indicate that exposure therapy can also be administered via virtual reality technology, namely *virtual reality exposure therapy *(VRET) [20,21]. VRET is a type of exposure therapy in which the user can be immersed into a computer-generated environment via a head-mount display and exposed to a simulation of a specific feared situation [20,21]. Contrary to other types of exposure therapy, VRET seems ideal for conditions where the real situations are inaccessible or costly (in vivo exposure) or individuals find it difficult to imagine various situations (imagined exposure therapy) [20,21]. To date, VRET has successfully been used for other forms of phobias, such as fear for spiders and flying, but has never been used in the treatment of fear of movement/exercise, nor pain catastrophizing in chronic pain conditions.

The previous postulation that imagined exposure therapy may effectively reduce pain catastrophizing in FMS patients makes the investigation of VRET as a treatment for pain catastrophizing in FMS a plausible option [17]. However, since no VRET program for the treatment of pain catastrophizing in FMS patients currently exists; preliminary steps are required prior to the development and testing of such a program. Initially, it has to be ascertained if visual exposure to catastrophized exercise activities cognitively triggers the functional brain areas associated with pain catastrophizing. The premise is that if visual stimuli of the catastrophized exercise activities cognitively trigger pain catastrophizing in the associated functional brain areas; graded and repeated exposure to visuals of the catastrophized exercise activities using a VRET program could possibly decrease pain catastrophizing and subsequently decrease fear to movement, essentially improving compliance towards exercise therapy programs among FMS patients. If the results of this proof-of-concept study are positive, this scientific breakthrough in FMS research will enable FMS sufferers to engage in beneficial physical activity without fear and possibly improve their quality of life. The following protocol presents the details of a project which aims to provide proof-of-concept for the further development and testing of a novel VRET program as treatment for exercise-related pain catastrophizing in FMS patients.

## Methods

Ethical approval for this study was obtained from the Health Research Ethics Committee of the Stellenbosch University during July 2010. Permission to conduct the study at the various study centres was granted by the various departmental heads. All eligible subjects will be required to read and sign an informed consent form before participating in this study. A unique study identification number/code will be allocated to subjects as they are entered into the study, thereby maintaining anonymity of all study participants. Confidentiality of participant's information will be maintained throughout the study process by storing the study data in a locked facility accessible only to the study personnel.

### Description of the overall project

The proposed project will consist of two interlinked experimental studies: Study 1 and Study 2.

**Study 1 **will incorporate an observational within-subject study design and aims to ascertain the neurophysiological changes occurring in the functional brain areas associated with pain catastrophizing when catastrophizing FMS patients are exposed to various visuals of exercise activities. Functional magnetic resonance imaging (fMRI) will be used to observe these neurophysiological changes within the associated functional brain areas. Study 1 will be conducted at the Cape Universities Brain Imaging Centre (CUBIC) situated at the Stellenbosch University's Tygerberg medical campus in Cape Town, South Africa (SA).

**Study 2 **will incorporate a quasi-, within-subject/between-groups experimental (pre- and post-, with/without treatment) study design and aims to ascertain the preliminary efficacy of visual exposure to exercise activities on pain catastrophizing in FMS patients. Clinical improvement in pain catastrophizing will be measured using objective and subjective outcome measures. The feasibility of using visual exposure to exercise activities as a treatment for pain catastrophization experienced by FMS patients in terms of: 1) time taken to administer; 2) ease of use; 3) safety of administration; 4) participant acceptability; and 5) participant opinion/thoughts of the intervention, will also be ascertained. Study 2 will be conducted at the Body in Motion analysis lab at the Stellenbosch University's Tygerberg medical campus in Cape Town, SA.

It is envisaged that the data collection period of the project will commence in June/July 2011 and be completed by September/October 2011.

### Study population and recruitment process

Twenty eligible subjects will be conveniently sampled from the available FMS population currently registered at the Tygerberg Hospital (TBH) Rheumatology clinic and/or attending the monthly FMS group meetings held at the TBH Occupational therapy (OT) department. The rheumatologists and/or occupational therapist working in the TBH Rheumatology clinic and/or OT department will be asked to identify all new or previously diagnosed FMS patients attending the clinic. The principle researcher and research assistant(s) will approach all potential patients personally or telephonically, and invite them to participate in the study. In addition, the clinic's patient database will be manually searched for all potential patients who have been diagnosed with FMS, but no longer attend the monthly meetings or the clinic. All potential patients listed on the database for whom contact details are available will be contacted telephonically and invited to participate in this project.

### Study outcomes

Study outcomes will include subjective and objective measures. Subjective measures will include pain catastrophizing, as measured using the Pain catastrophizing scale (PCS) and fear-avoidance behaviours/kinesiophobia, as measured using the Tampa scale of Kinesiophobia (TSK). The *PCS is a self-report measure which includes 13 items about the thoughts and feelings experienced when an individual is in pain. Respondents use a 5- point rating scale (where 0 = never to 4 = always), to indicate how often they experience each thought/feeling. The total score for the PCS equals 52, with a score above 24 indicating a high score. High internal reliability (alpha coefficient for total pain catastrophizing scale = 0.87) has been reported in patients with chronic pain with adequate validity and test-retest reliability *[12]. *The TSK is a self-report instrument designed to assess fear of pain and activity. It consists of 17 items each rated on a 4-point Likert scale. The total score for the TSK equals 68. Responses are summed to create a total score, with higher scores (above 37) indicating greater fear of pain and activity. The scale has demonstrated test-retest reliability and internal consistency (Cronbach alphas have ranged 0.68 to 0.80) in studies of patients with chronic low back pain. Stability over time and the criterion validity and construct validity has been previously established *[22].

Objective measures will include neurophysiological changes, as measured using the Siemens MAGNETOM Allegra 3 Tesla fMRI scanner available at the CUBIC facility. *The Allegra 3 Tesla fMRI scanner is a dedicated brain scanner and currently the most advanced brain imaging instrument on the market *[23].

### Subject inclusion and exclusion criteria

Adult male and female subjects aged 18 years and older, who have been clinically diagnosed with FMS according to the American College Rheumatology (ACR) criteria [24] by a qualified rheumatologist; subjectively display high PCS (more than 24 points) and TSK (more than 37 points) scores, and are registered as a patient at TBH Rheumatology clinic/OT department, will be included in this study. Subjects diagnosed with other conditions not related to FMS i.e. cancer; who have severe physical disabilities; who suffer from chronic rheumatoid conditions i.e. systemic lupus erythmatosus and rheumatoid arthritis; who have previously been hospitalized for a major psychiatric disorder; who have an uncontrolled endocrine or allergic disorder; who are using medication other than the prescribed pharmacologic agents for FMS symptoms; who have been using any narcotics long-term; who currently or who have previously abused any illicit substances or alcohol; have been diagnosed with epilepsy, or other conditions contraindicated in the use of visual exposures to stimuli; who display any contraindications which prohibit the use of fMRI, i.e. cardiac pacemakers, metal implants, claustrophobia, pregnancy and cochlear implants; and who are unable to discontinue intake of anti-depressants 4 weeks prior to commencement of study, will be excluded from this study.

### Allocation to study groups

On consenting to participate in this project, subjects will be randomly allocated into Group A (VRET/intervention group) or Group B ("waiting list"/control group) using a computer-generated random number sequence constructed by an independent statistician. Allocation will be concealed in opaque envelopes containing the letters 'A' or 'B' and administered by an independent individual not part of the study. Study procedures will be thoroughly explained to each subject. Sociodemographic forms, the General Practice Physical Activity Questionnaire (GPPAQ), and the Revised Fibromyalgia Impact Questionnaire (FIQR) will be administered to each subject at the beginning of study 1. *GPPAQ is a validated screening tool for use in primary care to assess adult (16 - 74 years) physical activity levels *[25]. *It provides a simple 4-level physical activity index, categorizing patients as inactive, moderately inactive, moderately active and active. The FIQR is an updated version of the Fibromyalgia Impact Questionnaire (FIQ) that has good psychometric properties, can be completed in less than 2 minutes and is easy to score. It has scoring characteristics comparable to the original FIQ, making it possible to compare past FIQR results with future FIQR results *[26]. *The FIQR assesses level of functioning. The original FIQ was developed and validated by Burckhardt et al (1991) to assess the current health status of women with FMS *[27].

Study groups will progress as a cluster group from study 1 to study 2 to reduce the period of time between baseline scans and final post-intervention/control scans.

### Study procedures

***Study 1: ***A neurophysiological analysis (fMRI scan) will be scheduled at a time most convenient for Group A and B subjects. Transport/remuneration for transport will be provided to and from the study venue on the day of the subject's scheduled appointment. On the day of the scheduled neurophysiological analysis, subjects will again be informed of the fMRI procedure and asked to comply with all the regulations of the laboratory. This preparation session will be conducted in the MRI simulation room situated within CUBIC facility. A set of the PCS and TSK will be administered prior to the commencement of the fMRI session and used as baseline data. Subjects will be escorted to the MRI room and asked to lie down inside the fMRI chamber. Foam cushions will be used to immobilize the head. The subjects will be required to wear MRI compatible earmuffs for communication with the experimenter and to minimize scanner noise. For structural localization, a MEMPRAGE structural sequence with a spatial resolution of *1 × 1 × 1 mm^3 ^*will be acquired (approx 9 minutes) for each subject. The tasks/stimuli used in the MRI sequence have been derived from other cognitive-behavioural therapy studies [15-17], but have been modified for application in this study. The tasks/stimuli have been specifically and carefully chosen and designed so as to elicit pain catastrophizing in the FMS subjects. The tasks/stimuli, although different, are also somewhat similar in concept/idea, so that they do not elicit completely different neurophysiological activity responses. The following tasks/stimuli will be applied during the fMRI scanning: 1) Visuals of exercise/physical activities (30s clips of various exercise activities) (4 minutes); 2) Visuals of everyday sedentary activities (i.e. 30s clip of reading a book/magazine) (4 minutes); and 3) Verbal visualization (30s of standardized verbal instruction where subject is instructed to imagine an activity i.e. running, cycling, skipping, etc.) (4 minutes). The fMRI sequence which entails the application of the three tasks/stimuli will last approximately 12 minutes and consist of 3 runs. The application of each of the three tasks/stimuli will constitute one run. Each run will last approximately 240 seconds (s) and comprises of: 4 × 30s 'off' periods (no stimulus) and 4 × 30s 'on' periods (stimuli). The order of the stimuli/'off' periods will be randomized using a coin-tossing procedure. In total, from preparation to completion, the MRI procedure for each subject will last approximately 40 minutes (15 minutes preparation + 9 minutes MEMPRAGE scan + 12 minutes MRI task ± 4 minutes extra).

***Study 2: ***On completing baseline scans in study 1, group B subjects (n = 10) will be instructed to complete another set of the PCS and TSK and continue daily activities as normal for the next eight weeks (control group). No other education/treatment/information will be given to group B subjects. Appointments to return to CUBIC for final scans after eight weeks will be scheduled for group B subjects. On completing baseline scans in study 1, one-hour long sessions will be scheduled for group A subjects (n = 10) twice a week for eight weeks (intervention group). During the twice weekly treatment sessions, subjects will be placed in an isolated room and be exposed to visuals of various exercise activities. Visual exposure to exercise activities will be delivered via a VR head-mount display (HMD); namely the eMagin Z800 3DVISOR linked to an ASUS K61IC series laptop. The eMagin Z800 3DVISOR has a highly sophisticated built-in head tracker which allows the user six degrees of freedom in motion [28]. The HMD is placed on the head of the user, blocking off the surrounding environment. The visuals relayed to the HMD via a connected laptop are viewed on two 3D OLED 0.59 inch micro displays, which to the user seem to play as huge as a 105-inch screen. These specific features of the eMagin Z800 3DVISOR enhance the immersion of the user into the virtual environment [28]. Software will consist of an hour-long DVD comprised of various simulated exercise activities. The subject may only be accompanied by a research assistant or translator. The PCS and TSK will be administered before and after each session. Information relating to the feasibility and logistics of the study will be collected from Group A's subjects at the end of the eight-week treatment program.

The PCS and TSK will be administered before and after the eight week time period for both groups. No PCS and TSK scores will be collected during the eight weeks for group B's subjects. At the end of the eight-week study period, both groups will undergo final fMRI scans.

#### • Feasibility and logistics

To determine the feasibility of the intervention and logistics of the procedure, the following information will be recorded using a datasheet which will include a post-treatment survey: time it takes to explain the procedure, equipment, visuals, etc. to the participant; time it takes the participant to familiarize himself with the equipment; time it takes to set-up the equipment; time it takes to administer the exposure session; any adverse effects due to intervention; ease of using the equipment; subject's acceptability and opinions/thoughts of using the equipment.

#### • Loss to follow-up

Should subjects not be able to handle being exposed to the visual of the exercise activities, he/she will be allowed to stop the exposure therapy treatment and carry on at a later stage. Should the subject refuse to participate any longer, data will be retained regardless as to how many sessions were completed and analyzed accordingly. All reasons for termination or suspension of treatments will be documented.

### Data collection and storage

The research assistant will document the randomized order of the stimuli/'off' periods during each run for each subject on a simple datasheet. This information will be blinded from the rest of the research team. Neurophysiological changes occurring during the fMRI sequence will be recorded and standard MEMPRAGE and EPI BOLD sequences will be acquired. fMRI data for each subject will be saved and stored on two hard-drives, under the reference number allocated to that subject. A research assistant will administer and collect completed PCS, TSK, GPPAQ and FIQR questionnaires, as well as sociodemographic forms from subjects. Data collected will be stored in the subject's respective folders. Back-up files of all data will be made and stored in a separate location. Folders, data and back-up data will be stored in a locked room with controlled access at all times. All subject identification and information will be kept confidential.

### Statistical analysis

***Study 1: ***fMRI data acquired from the subjects will be analyzed by a blinded radiologist or physiologist. The principle researcher will be involved in the analysis of the data. Contrasts between the different sets of data will be analyzed using Oxford's FMRIB Software Library (FSL), which is currently available at CUBIC. FSL is a collection of functional and structural brain image analysis tools, written by members of the Image Analysis Group at the Oxford Centre for Functional Magnetic Resonance Imaging of the Brain (FMRIB), Oxford University. FSL is distributed as freeware and most of the tools can be run both from the unix command line and as a graphical user interface [29]. Single-subject analyses will be performed by linear regression of the fMRI data. The functional brain regions to be analyzed and compared will include the areas previously associated with pain catastrophizing namely: the secondary somatosensory cortex (SII), contralateral insula, primary somatosensory cortex (SI), inferior parietal lobule and thalamus, contralateral anterior ACC, the contralateral and ipsilateral lentiform activation in SI, anterior and posterior cerebellum, posterior cingulate gyrus, and superior and inferior frontal gyrus [30]. The scans will be registered to high resolution Montreal Neurological Institute (MNI) structural scans so that the FSL grey matter atlas tools can be used to correctly locate the regions of interest.

***Study 2: ***Mean pre- and post-session (intervention group), as well as mean pre- and post-treatment (intervention and control group) PCS and TSK scores will be analyzed and compared between the groups and within-subjects for preliminary efficacy of the intervention. Paired *t test *for comparison of two dependent samples and contrast for the significance of the Pearson r correlation between two quantitative variables will be assessed. The open-ended questions of the post-treatment survey will be qualitatively analyzed and the closed-ended questions will be quantitatively analyzed using frequency counts. Post-intervention fMRI data will be analyzed and compared to subject fMRI data obtained at baseline. Single-subject analyses will be performed by linear regression of the fMRI data.

## Discussion

The premise is if visuals of various exercise activities trigger the functional brain areas associated with pain catastrophizing in FMS subjects; then as a treatment, it can be postulated that graded and repeated exposure to visuals of exercise activities using a VRET program could possibly decrease pain catastrophizing and subsequently decrease fear to movement, essentially improving compliance towards exercise therapy programs among FMS patients. Proof-of-concept for the development and testing of a novel VRET program for exercise-related pain catastrophizing in FMS will either be established or negated. The results of this project are envisaged to revolutionize FMS and pain catastrophizing research and in the future, assist health professionals and FMS patients in reducing despondency regarding FMS management.

## Competing interests

The authors declare that they have no competing interests.

## Authors' contributions

LDM conceptualized the project idea and study design and drafted the project proposal manuscript in preparation for publication submission. KAGS and QAL assisted in refining the project idea and study design and in drafting the final manuscript. BS provided the technical information relating to fMRI and assisted in refining the study design. All authors read and approved the final manuscript.

## Pre-publication history

The pre-publication history for this paper can be accessed here:

http://www.biomedcentral.com/1471-2474/12/85/prepub
